# Dengue fever outbreaks in Eritrea, 2005–2015: A case for strengthening surveillance, control and reporting

**DOI:** 10.1186/s41256-016-0016-5

**Published:** 2016-10-27

**Authors:** Abdulmumini Usman, Jacob D. Ball, Diana Patricia Rojas, Araia Berhane, Yohannes Ghebrat, Goitom Mebrahtu, Azmera Gebresellasie, Assefash Zehaie, Jacob Mufunda, Olivia Liseth, Ubydul Haque, Emmanuel Chanda

**Affiliations:** 1 WHO Eritrea Country Office, Asmara, Eritrea; 2 Department of Epidemiology, University of Florida, Gainesville, FL USA; 3 Emerging Pathogens Institute, University of Florida, 2055 Mowry Road, Gainesville, FL 32610 USA; 4 Division of Communicable Diseases Control, Ministry of Health, Asmara, Eritrea; 5 Ministry of Health, Asmara, Eritrea; 6 WHO Zambia Country Office, Lusaka, Zambia; 7 College of Engineering, University of Florida, Gainesville, FL USA; 8 Department of Geography, University of Florida, Gainesville, FL USA; 9 Vector Control Specialist/Consultant, Lusaka, Zambia

**Keywords:** Dengue fever, Capacity building, Surveillance, Vector control, Eritrea

## Abstract

**Background:**

The geographic distribution and burden of dengue is increasing globally. This study aims to evaluate dengue outbreaks and to substantiate the need for strengthened surveillance, reporting and control in Eritrea.

**Methods:**

Data from two cross-sectional dengue epidemic investigations in 2005 and 2010 were analyzed. Samples were tested for dengue virus-specific IgM and IgG antibodies using capture enzyme-linked immunosorbent assays. Dengue vectors’ breeding attributes were characterized and epidemic risk indices determined. National routine surveillance weekly reports from 2005 to the second quarter of 2015 were analyzed for spatiotemporal trends.

**Results:**

Dengue outbreaks increased in Eritrea from 2005 to 2015 with clinical presentation varying markedly among patients. The house and container indices for *Aedes aegypti* were 40 and 39.6 % respectively, with containers having *A. aeqypti* varying significantly (*P* < 0.04). Serum from 33.3 % (*n* = 15) and 88 % (*n* = 26) of clinical dengue cases in Aroget sub-Zoba (district) of Gash Barka Zoba (region) contained anti-DENV IgM antibody in 2005 and 2006, respectively. The national surveillance data from 2005 to 2015 indicate an overall spatiotemporal increase of dengue fever.

**Conclusions:**

The increase in dengue outbreaks has been confirmed in Eritrea and necessitates strengthening of surveillance and health worker and laboratory capacity, as well as targeted vector control interventions.

## Background

Dengue fever (DF) is a tropical vector-borne disease caused by four sero-types of a single positive-stranded RNA virus (DENV-1, DENV-2, DENV-3 and DENV-4) of the genus *Flavivirus,* [1]. The *Aedes aegypti* mosquito is the main vector for dengue fever, with *Aedes albopictus* being a secondary vector [2]. The infection has a wide range of symptoms ranging from completely asymptomatic disease through dengue fever and dengue haemorrhagic fever (DHF). Dengue fever is an acute febrile condition characterized by flu-like symptoms, and severe headache, retro-orbital pain, arthralgia and rash that affects infants, young children and adults, but seldom causes deaths. DHF is a severe and potentially deadly complication that involves plasma leakage that, in turn, causes dengue shock syndrome (DSS), fluid accumulation with respiratory distress, severe bleeding and extensive organ involvement [1, 2]. Mortality ranges from less than 1 % in dengue fever to about 26 % in DHF or DSS depending on the adequacy and timing of clinical care [1, 3].

Globally, 2.5 billion people live in dengue endemic areas, with an estimated 390 million infections occurring annually, and 100 million apparent infections [4–6]. Currently, no specific treatment for the disease exists, but DHF can be managed by rapidly administering fluid and plasma, and by monitoring vital signs [7]. Accordingly, vector control is presently the only method available for dengue control. Five vaccines are currently in development and one has completed phase III trials but no vaccine is approved yet [8]. This underscores the need to implement sustainable prevention and control interventions rather than rely on outbreak response and case management.

In Africa, where surveillance for dengue remains poor, epidemics have increased dramatically [9]. Major outbreaks have occurred in West Africa [10, 11], Southern Africa [9], and in countries along the eastern coast of the continent [12]. Equally, confirmed dengue fever outbreaks have been reported in Eritrea with the first confirmed outbreak in 2005 following the investigation of a suspected malaria outbreak [13]. There has been an increase in the magnitude and spread of the disease with new outbreaks in 2010 and 2015. The objective of this paper is to present and discuss dengue epidemiological data from Eritrea and findings from outbreak investigations during this time period within the context of recommended control strategies.

## Methods

### Ethical considerations

Ethical clearance for this study was sought from the Eritrean Ministry of Health’s Research Division’s Ethical Committee. Informed consent was sought from study participants, and assent was given by guardians for persons under the consenting age.

### Data collection

The case definition used in this study follows that issued by the Eritrean Ministry of Health, based on the World Health Organization from 1997 has been described in detail elsewhere [14]. At diagnosis, a dengue fever case is defined if a patient satisfies the following criteria: 1) Any person with acute febrile illness of 2–7 days duration with two or more of the following: headache, retro-orbital pain, myalgia, arthralgia, rash, haemorrhagic manifestations, leucopenia (probable case); a probable case with laboratory confirmation of one of the following: (positive IgM antibody, rise in IgG antibody titres, acute samples had positive RT-PCR or viral isolation) (confirmed case); a probable or confirmed case of dengue hemorrhagic fever (DHF); and all the above criteria, plus evidence of circulatory failure manifested by rapid and weak pulse, and narrow pulse pressure (≤20 mm Hg) or hypotension for age, cold, clammy skin and altered mental status (DSS). 2) Patient is from the affected areas. 3) Absence of malarial parasites in blood [15].

This study utilized two main approaches for data collection: analysis of two cross-sectional investigations of dengue outbreaks in 2005 and 2010 involving epidemiological and entomological approaches; the retrospective review of health management information system (HMIS) records from 2005 to 2015; and of integrated disease surveillance and response (IDSR) weekly reports from 2010 thru the second quarter of 2015.

### Epidemic investigations

The first outbreak of dengue fever was identified in Agordet sub-Zoba (district) of Gash Barka Zoba (region) in 2005 as a result of the investigation of a suspected malaria outbreak. The second outbreak was reported from Massawa sub-Zoba and surrounding areas in Semenawi Keith Bahri (SKB) Zoba in 2010 [16, 17]. Dengue fever outbreak investigations were conducted following the reports using clinical methods as well as laboratory serological confirmation of active human infection via viral isolation by cell culture or reverse transcriptase polymerase chain reaction (RT-PCR) from clinical samples for detection of DENV RNA. The detection of previous exposure to DENV was done through enzyme-linked immunosorbent assays (ELISA) to detect a minimum of a four-fold increase of IgG and IgM antibody titres to DENV.

#### Clinical presentation

Patients from the in-patient department (IPD) and outpatient department (OPD) at the time of the investigation were examined by a clinician and the symptoms were recorded in registers at Agordat hospital and the private clinics. The hospital registers were reviewed retrospectively and analyzed for spatial and temporal trends. Case-based outbreak investigation forms were completed for dengue cases at the OPD and those at IPD in the Zonal Referral Hospital of Massawa and Ghedem hospital. Only suspected dengue fever cases that satisfied the inclusion criteria outlined above were interviewed, examined and had blood drawn for serological laboratory analysis using ELISA.

#### Serological/Laboratory confirmation

Blood samples were collected from suspected dengue cases and processed for serological screening after ruling out malaria infection through the blood film test or rapid diagnostic test (15 samples from Agordet and 26 samples from Massawa). Acute and convalescent sera were transported on cold chain to the National Health Laboratory in Asmara and couriered to collaborating institutions in Kenya for further analysis. Panbio Dengue IgM antibody capture enzyme-linked immunosorbent assays (MAC-ELISA) were used per manufacturer instructions to detect anti-DENV IgM antibodies in convalescent samples [18]. The IgG-ELISA protocol has been previously described [19], and was performed to detect anti-DENV IgG antibodies (the threshold for which was set at a 4-fold increase).

#### Entomological investigation

Standard entomological techniques were harnessed for surveillance of immature and adult stages of *Ae. aegypti* in the highly affected area of Agordet. All potential mosquito breeding containers in domestic and peri-domestic areas of dwellings were searched for larvae and pupae. Breeding sites characteristics, household and container indices, proportion of containers positive for *Ae. aegypti*, and the epidemic risk indices were determined. Potential indoor and outdoor resting places were also search for adult mosquitoes. Adult and larval morphological identifications were done in Asmara using appropriate taxonomic keys [20].

#### The epidemic threshold and magnitude

**Fig. 1:**
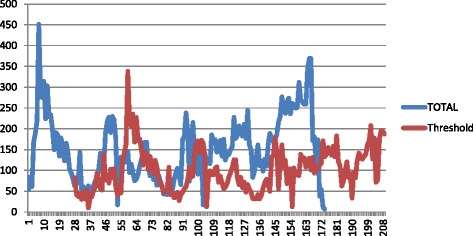
National Weekly Dengue Cases and Epidemic Threshold 2012–2015. Here blue indicate total and red threshold

**Fig. 2:**
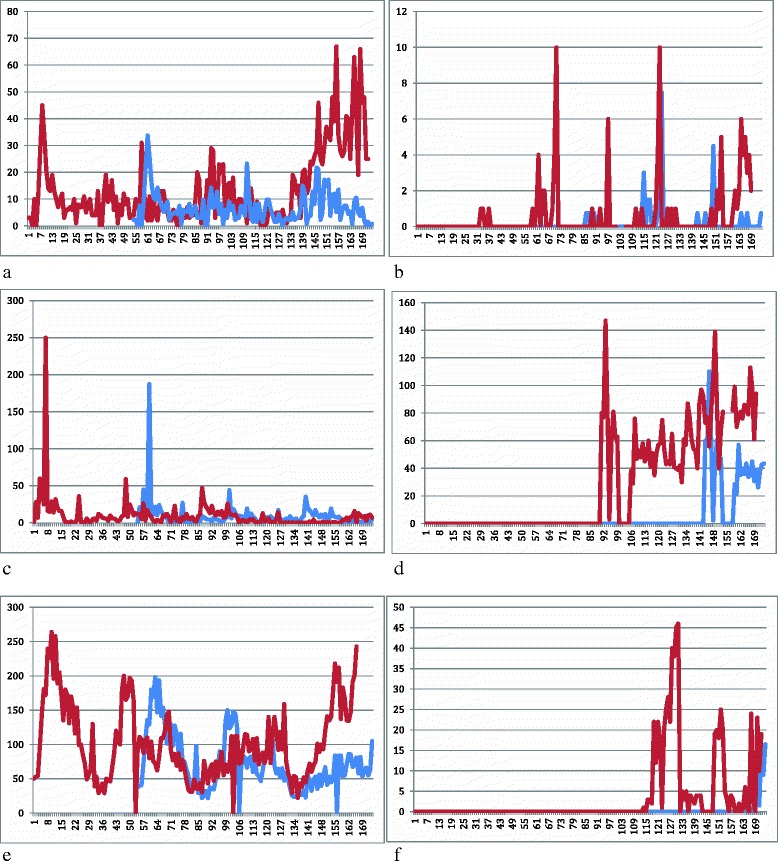
Weekly Dengue Cases (red line) and Epidemic Threshold (blue line) 2012–2015 by Zoba. **a** is Anseba, **b** is Debub, **c** is Maekel, **d** is G/Barka, **e** is NRS and **f** is SRS

The weekly IDSR data from 2012 to 2015 are recorded in an Excel database and epidemic alert thresholds for the IDSR data from 2012 to 2015 are calculated based on the methodology proposed for malaria [21]. The weekly epidemic alert threshold is calculated when cases exceed the 75^th^ percentile of the previous year’s weekly cases for each Zoba and National level. National weekly trend graphs are plotted for the entire time series along with the epidemic threshold (Fig. 1), and then broken down by Zoba (Fig. 2a-f).

### Review of IDSR/HMIS reports

The Eritrean Ministry of Health has been operating an extensive HMIS database in the central registry since 1998; the HMIS also captures IDSR data. Thus, complete data for dengue is routinely collected at health facilities and reported thru the IDSR system [15]. The data were analyzed to describe the occurrence of dengue virus-symptomatic individuals in absolute numbers and percentage by place (residence, travel, type of work) and demographic (age, sex, occupation) characteristics.

## Results

### Epidemiological investigations

#### Clinical presentation

At Agordet, clinical presentation after fever varied markedly among the 1142 dengue patients; from headache (70 %), arthralgia and back pain (41 %), bleeding in the form of epistaxis (2 %) and myalgia, lymphadenopathy and retro-orbital pain manifested in 20, 18 and 15 % of the dengue patients, respectively. At Massawa, out of the suspected 26 dengue fever cases, 11 (42 %) were from Ghedem Hospital, while the remaining 15 (58 %) were from Massawa Hospital. All dengue patients presented typical signs and symptoms of dengue fever: high fever, headache, fatigue, muscular or joint pain and anorexia; none had any signs of bleeding or other hemorrhagic tendencies. 88 % of all dengue patients were permanent residents of Massawa sub-Zoba (23/26), while one was from Debub Zoba and two were from Maakel Zoba. The distribution of the dengue patients by occupation varied from soldiers 6/26 (23 %), to students 4/26 (15 %) and construction workers 3/26 (12 %).

### Epidemic threshold

In Agordet, the third quartile threshold for malaria epidemic reporting was crossed in week 8 of the year and triggered the investigation and confirmation of the dengue outbreak in week 13 of 2005 (Appendix 1). At Massawa in 2009, the peak incidence of suspected dengue cases in the Zoba was initially seen in the 9th and 12th weeks, which then slightly declined and peaked in week 15, 16 and 19. Then the incidence declined gradually until the 28th week with no cases (Appendix 2). In 2010, the highest numbers of cases (211) were recorded in Week 9. However, there were no admissions of suspected cases, no referrals and no deaths in the hospital.

### Magnitude

Records of 1132 dengue symptomatic cases were traced during the period of the outbreak at Agordat hospital and the private clinics. The outbreak started during week 3 of the year and continued up to week 27 (Appendix 3). All age groups were affected but the attack rate was highest from 20 to 40 years and males were more affected than females. At the Zonal Referral Hospital of Massawa and Ghedem hospitals, only 19 % of the dengue cases were females and the age group ranged from 9 to 58 years. The outbreak started in November 2009 and continued till March 2010. No deaths were recorded in either outbreak investigation.

#### Entomological surveys

*Ae. aegypti,* was identified in Agordet with high epidemic risk indices according to both the Breteau Index and house index. The house and container indices were 40 and 39.6 % respectively. Container positivity for *Ae. aegypti* varied significantly from cement tanks (85.3 %) and clay pots (33.3 %) to plastic tanks (10 %) and iron barrels (4 %) (*P* < 0.035). At Massawa, breeding sites included seeping water from public tap water, marshy areas, and broken pipes with uncountable larval densities.

#### Serological/Laboratory confirmation

Seropositivity to DENV has been demonstrated in two geographically segregated places in Eritrea. In 2005, seropositivity to IgM antibodies to dengue viruses was 33.3 % (*n* = 15) at Agordet sub-Zoba of Gash Barka Zoba. In 2010, sero-positivity for DENV-1 was 88 % (*n* = 26) at Massawa sub-Zoba and surrounding areas in SKB Zoba.

### Review of IDSR/HMIS reports

Data from the HMIS indicate large outbreaks of dengue fever in 2005 and 2009 in SKB Zoba. The total number of suspected dengue cases reported increased seven-fold from 328 in 2008 to 2160 cases in 2009. The attack rate in 2009 was highest in Ghindae and Massawa, 1513 and 978 per 100,000 people respectively. Outbreaks were reported in Keren and Anseba Zobas in 2014 and in Mendefera and Debub Zobas in 2015 (Table 1). Age and sex-specific attack rates show that dengue had the highest burden on persons aged 20-39, and that children under 5 face a substantial burden of disease which could be linked to child mortality (Table 2).

**Table 1:** Population and dengue fever cases by year and Zobas in Eritrea

	Dengue fever cases							
Year	Anseba	Debub	DKB	Gash Barka	Maekel	NRS	SRS	National
2005	214	28	210	19	239	–	3771	4481
2006	11	–	72	8	28	–	269	388
2007	–	–	44	–	266	3	349	662
2008	1	–	70	880	5	–	328	1284
2009	–	–	407	378	11	1	2150	2947
2010	1101	31	366	3414	298	11	7725	12,946
2011	742	23	1727	5405	461	51	5786	14,195
2012	948	46	953	4343	545	318	5786	12,939
2013	695	38	1008	6777	424	305	3256	12,503
2014	852	54	711	5451	56	184	3581	10,889
2015 (Jan–June)	493	164	232	2555	235	241	2951	6871
Population 2015	583,434	953,497	83,004	867,818	671,390	1114	440,241	3,599,384

**Table 2:** Age and sex-specific attack rates

Age group	Attack rate/1000		
	Males	Females	Total
<5	44.75	34.95	40.00
5–9	17.48	19.19	22.55
10–19	39.42	27.09	33.09
20–29	92.61	41.03	56.51
30–39	86.79	37.99	52.27
40–49	63.70	20.84	37.19
50+	22.97	13.68	17.99
Total	42.49	27.60	34.30

#### Spread

National IDSR/HMIS data from 2010 to 2015 indicate an overall increase in spatial and temporal trends of the disease (Table 1, Figs. 1 and 2a-f).

## Discussion

**Fig. 3:**
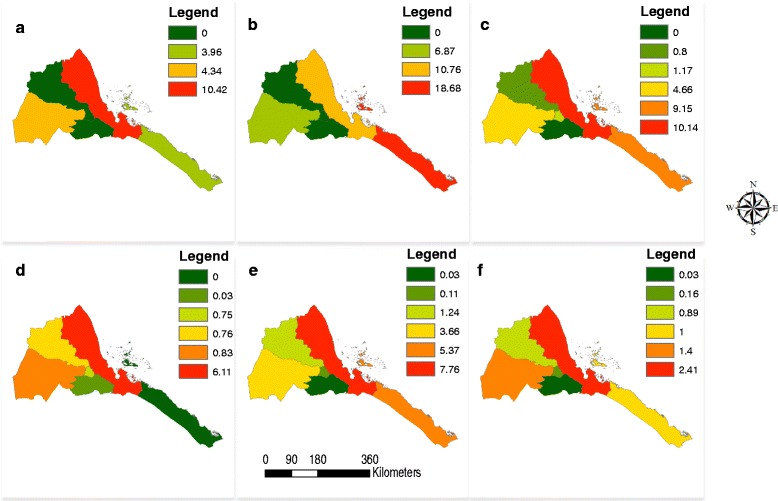
The spatial and temporal distribution of Dengue Fever in Eritrea 2010–2015. **a** = 2010; **b** = 2011; **c** = 2012; **d** = 2013; **e** = 2014; **f** = 2015

Dengue fever is an epidemic prone disease that is rapidly expanding globally [22] and in Eritrea, as evidenced by the results of the present study. This study is the first to describe the clinical features of suspected, probable and confirmed dengue cases, and to confirm dengue epidemics in Eritrea. The analysis of the IDSR/HMIS data indicates a rapid spatial and temporal spread of the disease to almost the entire country (Table 1 and Fig. 3). The most common *Ae. aegypti* breeding sites in the study areas were cement tanks (85.3 %) and clay pots (33.3 %) which need to be targeted for an effective control of immature stages of the vector. Furthermore, house and container indices indicated that *Ae. aegypti* is widespread among cement tanks and clay pots, indicating that targeting peri-domestic and domestic breeding sites with larvicide and other environmental management interventions may be effective in reducing the burden of dengue in Eritrea [23, 24]. Integrated vector control strategies have been shown to be effective at reducing the burden of dengue in other endemic countries such as Thailand (e.g. [25], Vietnam e.g. [26]), Costa Rick, Egypt and Kenya [27], may also be effective in Eritrea.

Despite the existence of potentially effective interventions, technical capacity for health workers to control dengue outbreaks remains a notable challenge in Eritrea. The 2005 dengue outbreak was reported to the central IDSR unit 10 weeks after its onset, only after failing to confirm a malaria outbreak [17]. This late reporting is not uncommon as dengue diagnostic criteria are non-specific and approximately 70 % of all infections are asymptomatic [28]. Thus, the epidemics described here provide a conservative estimate of the true burden and distribution of dengue in Eritrea. Though there were no cases of dengue mortality reported in Eritrea, it is probable that dengue in Eritrea may be underreported for various reasons including low awareness of the disease among health providers, poor surveillance, possible misdiagnosis due to overlap with malaria cases and lack of relevant systems for definitive diagnosis at health stations [21]. The lack of an in-country laboratory to confirm dengue serotypes underscores the need to strengthen public health infrastructure in Eritrea.

Data on the spatial and temporal distribution, abundance and ecology of *Ae. aegypti* in Eritrea remain sparse and there has not been a surveillance system that would map the areas at risk of dengue in Eritrea. While the numbers of containers with *Ae. aegypti* larvae and eggs during this entomological survey for dengue were low, the house and container indices were higher than the recommended thresholds of 3 and 4 %, respectively, indicating a potential epidemic risk [29]. Household and container epidemic risk indices have been shown to not be useful for assessing risk of infection as they do not consider factors such as population density, temperature, human habits and population immunity [30, 31]. Studies have demonstrated that dengue virus infection rates are temperature-dependent, demonstrating increased infection and transmission rates at higher temperatures until a threshold temperature is surpassed and transmission decreases [32–36]. This could partially explain the observed spatial spread of dengue across Eritrea, where average temperatures are usually high and have minimal fluctuations. However, in Eritrea there seems to be an association between altitude and dengue fever; previous outbreaks were in Agordat Zoba, 604 m above sea level (masl), Massawa Zoba, 20 masl, and in Ghindae Zoba, 915 masl. Recent outbreaks in 2014 in Keren Zoba, which is 1422 masl, and in 2015 in Mendefera Zoba, which is 1954 masl. may indicate ecological niche expansion and an increased risk of infection for the densely populated highland communities in Eritrea.

To further explore the association between environmental factors and dengue cases and identify the high risk areas for dengue transmission in Eritrea, IDSR data and entomological surveillance data including information from cross sectional surveys should be correlated with environmental data at Zoba, sub-Zoba and community levels. This will optimize the use of the limited resources available for dengue fever prevention and control, facilitate identification of high-risk areas, and increase the effectiveness of interventions. Eritrea should conduct a seroprevalence survey in a random sample of the population to determine which serotypes are circulating and to identify relevant correlates of infection.

This study demonstrates spatiotemporal spread of dengue in Eritrea during 2005–2015 indicating that preventative strategies for dengue fever and other potential disease vectored by *Aedes aegypti* should be strengthened to achieve the most cost-effective outcomes [17, 21]. The data presented here are limited and subject to reporting biases mentioned above, which are related to Eritrea’s limited dengue surveillance infrastructure. As such, our results are descriptive because statistical analyses would not reliably quantify spread. Furthermore, cross-reaction of DENV with other arboviruses is a problem that could have influenced our results—there could be false positive dengue cases where the patient was in fact infected by another arbovirus. Additionally, rapid tests were designed for outbreaks, and the application of these tests in serosurveys may change the reliability of the tests.

Expanding preventative measures should be coupled with extensive entomological surveillance to have a sustained impact on dengue burden.

## Conclusion

There is a notable increase in dengue outbreaks in Eritrea, which necessitates strengthening of surveillance, health worker and laboratory capacity as well as targeted interventions including vector control. This would allow for faster, more efficient outbreak responses, and improved health outcomes for dengue patients. Increased capacity for vector control would allow for the development, implementation, and then monitoring and evaluation of integrated vector control strategies and community education campaigns to reduce the vector population.

## Figures and Tables

**Fig. 4:**
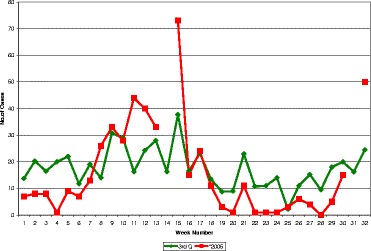
Weekly Malaria trend for Agordet Hospital

**Fig. 5:**
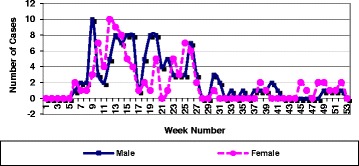
The peak incidence of suspected dengue Fever By gender in the SKB Zone in 2009

**Fig. 6:**
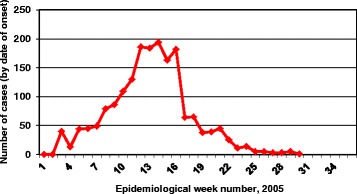
Weekly Distribution of dengue cases at Agordet Hospital
